# Regulation of Pre-Osteoblasts Seeded onto Titanium and Zirconia Through Modification by Hydrofluoric Acid and LPS Challenge

**DOI:** 10.3390/dj14060378

**Published:** 2026-06-18

**Authors:** Joao Moura Neto, Larrisa M. S. C. Raucci, Ana Carolina Chagas, Mariana Ferreira Caraschi, Isabela Massaro Ribeiro, Taisa Nogueira Pansani, Carlos Alberto de Souza Costa, Fernanda Gonçalves Basso

**Affiliations:** 1 Dental School, Ribeirão Preto University (UNAERP), Ribeirão Preto14096-900, Brazill.raucci@unaerp.br (L.M.S.C.R.);; 2 Araraquara Dental School, São Paulo State University (Unesp), Araraquara 14801-903, Brazil; acchagas@unesp.br (A.C.C.); mcaraschi@unesp.br (M.F.C.); taisa.n.pansani@unesp.br (T.N.P.); cas.costa@unesp.br (C.A.d.S.C.)

**Keywords:** implant, titanium, zirconia, LPS

## Abstract

Background/Objectives: Surface modifications of implants aim to mimic bone tissue and provide a more suitable environment for cell metabolism. Several modifications have been proposed, and in addition to evaluating the effects of these treatments on cell behaviour, it is also essential to determine the response of these cells to an inflammatory environment. This investigation evaluated the behaviour of murine pre-osteoblasts seeded onto acid-treated titanium and zirconia surfaces subjected to inflammatory challenge. Methods: Discs were manually polished using abrasive paper and then subjected to surface modification by hydrofluoric acid through distinct protocols according to each material. Surface topography and roughness were determined using scanning electron microscopy (SEM) and ImageJ software (Version 2.16). Then, MC3T3 cells were seeded onto the discs for 24 h and subsequently exposed to lipopolysaccharides (LPSs) from *Porphyromonas gingivalis* (*P. gingivalis*) (1 μg/mL) for 4 h at 37 °C. The cells were then evaluated for viability, oxidative response, and gene expression of pro-inflammatory cytokines. Results and Conclusions: Both materials were affected by acid treatment, resulting in more irregular topography and increased surface roughness.

## 1. Introduction

One of the main factors affecting the osseointegration process is implant material. Titanium is the gold-standard material for osseointegrated implants; however, ceramic implants obtained from zirconia are also increasingly being applied for oral rehabilitation [1]. Pre-clinical and clinical investigations demonstrated that ceramic implants show better colour characteristics when compared to titanium, as well as biocompatibility, lower microorganism affinity, lower corrosion, and ionic release, in addition to a lower inflammatory and immunogenic response [1]. These characteristics may lead to accelerated cell attachment and higher capacity to respond to inflammatory stimuli.

However, despite these characteristics, zirconia’s high smoothness may hamper the interactions of cells with these surfaces [2]. For this reason, the application of zirconia in implants requires surface modifications.

Several investigations demonstrated that surface modifications of titanium result in higher surface roughness, which leads to better interactions with bone cells and faster osseointegration [3]. Recently, a consensus was reached on the concept of modified titanium implants showing superior performance to polished discs in terms of bone cell interactions and metabolism [3]. These events are related to an increased contact surface between implants and receptor tissue, favouring cell adhesion and deposition of the extracellular matrix [3]. Epigenetic investigations demonstrated that these modifications trigger and accelerate the expression of osteogenic markers such as collagen, RUNX2, and alkaline phosphatase, leading to the acceleration of bone formation [4,5].

In terms of modifications, we may cite acid treatment, sandblasting, additive manufacture, and biofunctionalization, among others [6]. However, notwithstanding the positive results obtained from in vitro and in vivo studies, the majority of data on this topic were obtained using aseptic/non-inflammatory environment models, which do not fully represent peri-implant conditions. The resolution or prolongation of the inflammatory environment around an implant is a primary factor impacting peri-implant repair. Some investigations demonstrate that improved interactions of cells with implant surfaces may induce phenotypic changes that modulate the inflammatory response, decreasing synthesis of pro-inflammatory cytokines by these cells and accelerating the resolution of this phase, which triggers the transition to subsequent phases of tissue repair [7]. Against this background, this study evaluated the effect of surface acid modifications of titanium and zirconia discs on pre-osteoblast response in the presence of an inflammatory challenge.

## 2. Material and Methods

### 2.1. Zirconia and Titanium Discs

Zirconia discs (2 mm thick and 8 mm in diameter) were obtained from cylinders cut from ICE ZIRKON transluzente plus (Zirkonzahn, Gais, Italy) zirconia blocks using a precision cutter (ISOMET 1000, Buehler, Lake Bluff, IL, USA) [8].

Titanium discs (2 mm thick and 8 mm in diameter) were obtained through machining of a commercially pure grade 4 titanium cylinder.

Initial surface roughness was standardized through manual polishing using abrasive paper (400, 600, 1200 and 2000) (T469-SF- Noton, Saint-Gobam Abrasivos Ltd.a., Jundiaí, SP, Brazil), followed by a washing procedure with ethanol, acetone, and water in an ultrasonic bath [8].

Surface modification was performed using hydrofluoridric acid (HF) treatment; for zirconia, this entailed immersion in 40% HF solution for 2 h under agitation at 25 °C, while for titanium, it entailed immersion in 10% HF solution for 5 min.

### 2.2. Surface Characterization

Surface topography and roughness were analyzed using images obtained from scanning electron microscopy (SEM; Inspect Scanning Electron Microscope-S50, FEI, Hillsboro, OR, USA). Acquired images were analyzed by means of ImageJ software (Version 2.16) (National Institute of Health—NIH, Bethesda, MD, USA) in order to obtain mean surface roughness through the measurement of Ra of five distinct linear areas. Surface plots were acquired by selecting the central area of each sample.

### 2.3. Cell Culture

MC3T3 pre-osteoblasts were cultured in alpha-MEM culture medium (Gibco, Carlsbadl, CA, USA) supplemented with 1% antibiotic solution (PenStrep, Gibco) and 10% fetal bovine serum (Gibco). They were subcultured through enzymatic disaggregation using 0.25% trypsin (Gibco).

Discs (polished or acid-treated) were placed in 24-well plates and stabilized using metallic devices. Then, cells were cultured onto the discs (3 × 10^4^ cells/disc) in 1 mL of complete culture medium for 24 h.

### 2.4. LPS Challenge

To simulate inflammatory conditions, cells were challenged with lipopolysaccharides from *Porphyromonas gingivalis (P. gingivalis)* at 1 ug/mL (Sigma-Aldrich, St Louis, MO, USA). LPS was added to the cells after 24 h of seeding on discs and maintained for another 4 h. After this period, phenotypic characteristics were analyzed.

### 2.5. Analysis of Cell Viability

Cell viability was determined using the PrestoBlue assay (Invitrogen, Carlsbad, CA, USA), which demonstrates the conversion of resazurin salt into resorufin, a fluorescent dye. For this purpose, cells were incubated in a 10% PrestoBlue solution in SFB-free DMEM for 1 h at 37 °C and 5% CO_2_. Fluorescence intensity was measured by a fluorimeter at 560/590 nm (Synergy H1 Hybrid Multi-mode Microplate Reader, Biotek, Winooski, VT, USA).

### 2.6. Oxidative Response

Cell oxidative response was determined by evaluating synthesis of reactive oxygen species using an NO-synthesis kit (Sigma-Aldrich). Fifty microliters of the culture medium that remained in contact with cells during treatments was collected and added to a 96-well plate. Then, 50 μL of Griess reagent was added to the samples, followed by incubation for 10 min in the dark. The resulting solution was analyzed using a spectrophotometer at 540 nm (Synergy H1).

### 2.7. Gene Expression of Pro-Inflammatory Mediators

Gene expression of pro-inflammatory cytokines was determined through qPCR. For this assay, cells were exposed to *P. gingivalis* LPS (1 μg/mL) for 4 h. Total RNA was isolated by a PureLink RNA MiniKit (Invitrogen), and cDNA was obtained using a High Capacity cDNA Reverse Transcriptions Kit (Applied Biosystems, Foster City, CA, USA).

For PCRs, SyberGreen Universal PCR Master Mix and specific primers were applied. Fluorescence was analyzed using Step One Plus (Applied Biosystems), and the results were normalized by GAPDH gene expression.

### 2.8. Statistical Analysis

Qualitative data were descriptively presented, while quantitative data were analyzed by ANOVA and Tukey tests at a 5% significance level.

## 3. Results

### 3.1. Surface Roughness and Topography

**Figure 1:**
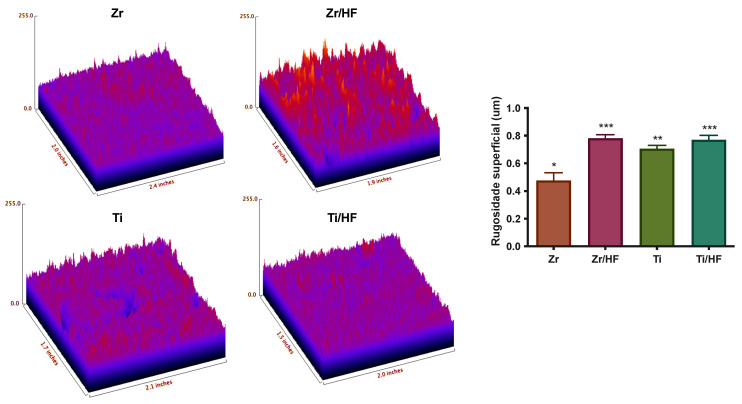
Surface topography and roughness, demonstrating different amplitudes of peaks and valleys for different surfaces. The graph demonstrates mean values for surface roughness for each group. Distinct symbols identifying groups indicate statistically significant differences (Tukey; *p* < 0.05). Brighter colors indicate higher peaks.

Surface modification with hydrofluoridric acid resulted in increased roughness for both materials, with no difference between them; however, this effect was more evident for zirconia discs relative to initial roughness (Figure 1).

### 3.2. Cell Viability

**Figure 2:**
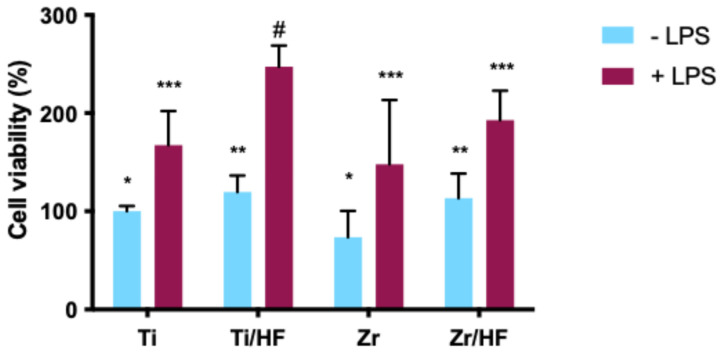
Viability of osteoblasts seeded onto different surfaces, with or without subsequent LPS treatment. Bars indicate mean values of each group. Groups identified by distinct symbols are statistically different. +LPS-presence of LPS/-LPS-absence of LPS.

In the absence of LPS, similar viability was observed for pre-osteoblasts seeded onto Ti and Zr discs (Figure 2). HF-treated surfaces were associated with increased cell viability, with no difference between discs. The LPS stimulus further resulted in increased cell viability in pre-osteoblasts, especially for Ti/HF discs.

### 3.3. NO Production

**Figure 3:**
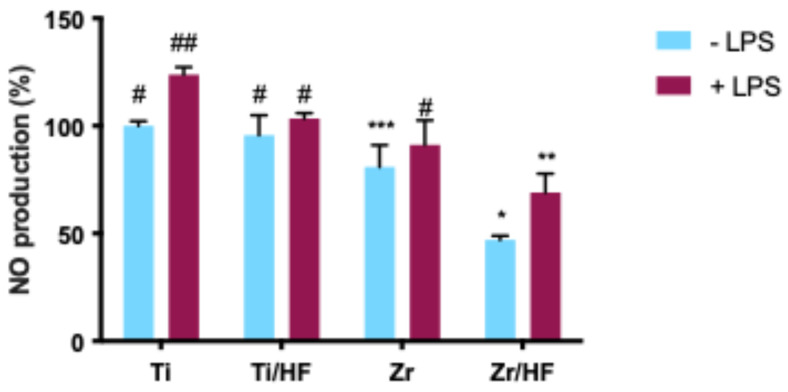
NO production by osteoblasts seeded onto different surfaces, with or without subsequent LPS treatment. Bars indicate mean values of each group. Groups identified by distinct symbols are statistically different.

The lowest NO synthesis values were detected for Zr discs in the absence of the LPS stimulus (Figure 3), while the highest values were detected for Ti discs. Again, oxidative response was also increased for cells exposed to the LPS stimulus, and the highest levels were observed for Ti discs (Figure 3).

### 3.4. Gene Expression of Pro-Inflammatory Cytokines

**Figure 4:**
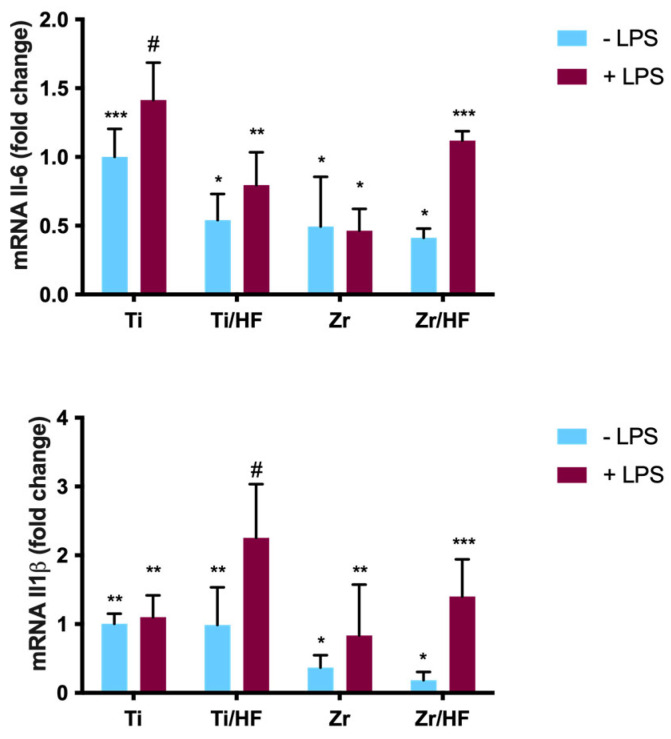
Gene expression of IL1β and IL-6 in osteoblasts seeded onto different surfaces, with or without subsequent LPS treatment. Bars indicate mean values of each group. Groups identified by distinct symbols are statistically different.

Gene expression of IL1β was higher in the Ti and Ti/HF groups compared to the Zr and Zr/HF groups, even in the absence of the LPS stimulus (Figure 4). After LPS was added to the cell culture, synthesis of IL1β increased for all groups except the Ti group.

Synthesis of IL-6 demonstrated a distinct pattern of response, where the Ti group showed the highest rates of synthesis compared to the other groups in the absence of LPS. In the presence of the inflammatory stimulus, synthesis of IL-6 increased, except in the Zr group.

## 4. Discussion

Osseointegration of oral implants depends on several factors, such as interaction of osteoblasts with implant surfaces and the deposition and maturation of extracellular matrix by these cells [9,10].

In this regard, to improve the adhesion and metabolism of osteoblasts on these surfaces, several surface modifications have been investigated, such as sandblasting, surface alkalinization, and acid treatment [11,12]. These modifications aim to increase surface roughness through the alteration of topography, creating micro- and nanomodifications that facilitate the adhesion and spread of osteoblasts [13]. These morphological changes lead to higher cell metabolism, demonstrated by increased collagen synthesis and mineralization [13].

Another factor directly related to cell behaviour during peri-implant repair is the type of material. For several years, titanium has been considered a gold-standard option; however, ceramic materials such as zirconia have also emerged in this context, demonstrating mechanical stability and satisfactory biological response [9,10,14].

The present investigation demonstrated the effect of surface modification of titanium and zirconia discs on osteoblast viability and metabolism.

At first, an increase in surface roughness was demonstrated by SEM, corroborated by graphical topography results. Without surface modification, Zr discs presented lower values, as previously demonstrated [14]. After HF treatment, however, the ceramic material showed higher rates of surface roughness. These results are consistent with cell viability analyses, where higher rates are demonstrated for HF-treated discs [11,13].

Several investigations have already demonstrated a direct relationship between surface topography and the adhesion of mesenchymal cells, especially osteoblasts, to substrates [11]. Particularly for titanium surfaces, higher surface roughness is associated with increased cell adhesion and differentiation [3,13]. Zirconia implants, however, have shown lower rates of cell attachment, especially due to decreased roughness [14]. Therefore, in this case surface modification is mandatory and significantly improves the suitability of this material for osseointegrated implants [12].

In the presence of an inflammatory stimulus (LPS), all groups demonstrated increased cell viability, which may be associated with its low/moderate intensity, as previously demonstrated [8,15].

In the absence of LPS, the lowest rates of NO production were found for Zr discs, while the highest levels were detected for cells seeded onto polished Ti discs after LPS treatment. These results may be correlated with the lower inflammatory potential of the former material, as well as with the higher attachment of cells onto this surface, which improves the responsiveness and tolerance of cells to inflammatory stimuli [8,13].

Gene expression of inflammatory markers was increased after LPS challenge, which may reflect the inflammatory potential of both materials. Cells exposed to LPS seeded onto Ti surfaces showed higher expression of inflammatory mediators when compared to those exposed to LPS-treated Zr surfaces. However, contrary behaviours were observed for both surfaces. Ti-modified surfaces showed a decrease in LPS response, while cells seeded onto Zr surfaces demonstrated increased expression of inflammatory mediators. This may be due to several factors, such as improved cell adhesion onto Zr surfaces, which may lead to a distinct phenotype [8,13].

In conclusion, despite its lower inflammatory potential, the characteristics of polished Zr may represent a challenge to cell adhesion and synthesis. On the other hand, a surface treatment such as HF may be a suitable model for modulating peri-implant repair.

## Data Availability

The data presented in this study are available on request from the corresponding author. The data are not publicly available due to privacy restrictions.
